# Primary Molecular Disorders and Secondary Biological Adaptations in Bartter Syndrome

**DOI:** 10.4061/2011/396209

**Published:** 2011-09-20

**Authors:** Georges Deschênes, Marc Fila

**Affiliations:** ^1^Pediatric Nephrology Unit, Hôpital Robert-Debré, 48 Bd Sérurier, 75019 Paris, France; ^2^Faculté de Médecine Xavier Bichat, University Paris 7, 16 rue Henri Huchard, 75018 Paris, France

## Abstract

Bartter syndrome is a hereditary disorder that has been characterized by the association of hypokalemia, alkalosis, and the hypertrophy of the juxtaglomerular complex with secondary hyperaldosteronism and normal blood pressure. By contrast, the genetic causes of Bartter syndrome primarily affect molecular structures directly involved in the sodium reabsorption at the level of the Henle loop. The ensuing urinary sodium wasting and chronic sodium depletion are responsible for the contraction of the extracellular volume, the activation of the renin-aldosterone axis, the secretion of prostaglandins, and the biological adaptations of downstream tubular segments, meaning the distal convoluted tubule and the collecting duct. These secondary biological adaptations lead to hypokalemia and alkalosis, illustrating a close integration of the solutes regulation in the tubular structures.

## 1. Introduction

**Figure 1:**
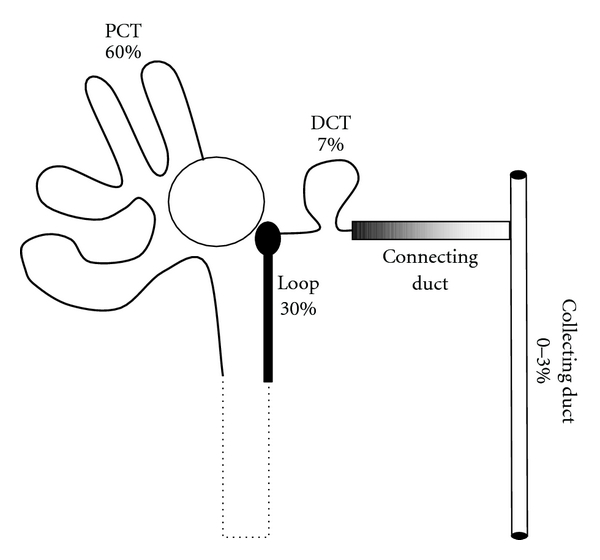
*Segmentation of sodium reabsorption*. Sodium reabsorption in tubular epithelial cells proceeds along a general two-step mechanism that includes a/an active extrusion of intracellular sodium ions by the basolateral sodium pump that is common to all tubular segments, b/ a passive apical entry of sodium dissipating the electrochemical gradient generated by the sodium pump via an exchanger or a cotransporter or a sodium channel that is specific to each tubular segment. Briefly, 175 L/1.73 m² of plasma roughly containing 20 moles of sodium chloride are filtered every day by the glomeruli, and 99 to 100% of this amount is reabsorbed in the convoluted and straight tubules (60%), the thick ascending limb of the Henle loop (30%), the distal convoluted tubule (7%), the connecting and the collecting duct (0 to 3%, controlled by aldosterone and angiotensin-2). The thin descending and ascending limbs of the Henle loop do not display any capacities to reabsorb sodium. Proximal tubular failure leads to the Fanconi syndrome where sodium wasting is associated with numerous solute wasting (potassium, bicarbonates, calcium, phosphates, glucose, aminoacids). Failure of the large ascending limb of the Henle loop is responsible for the Bartter syndrome, of the distal convoluted tubule for the Gitelman syndrome, and of the collecting duct for type 1 Pseudohypoaldosteronism [3].

**Figure 2:**
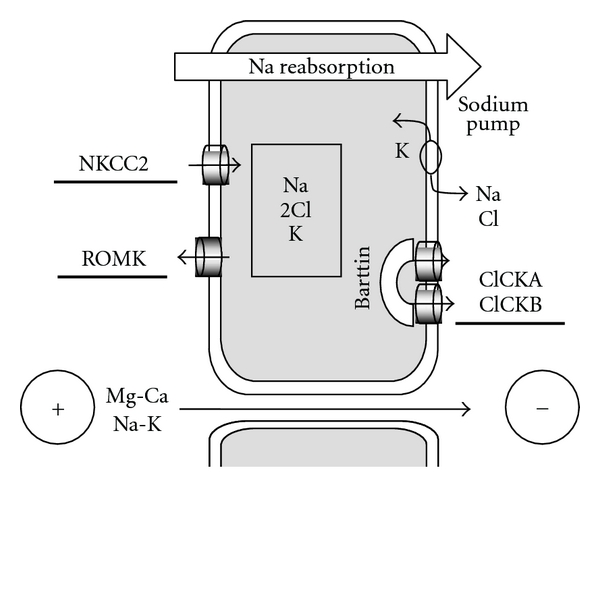
*Structures involved in sodium reabsorption in the Henle loop.* The large part of the Henle loop is mainly dedicated to sodium reabsorption. The system is vectorized and energized by the sodium pump at the basolateral face of the cell. KCNJ10 (not drawn on the figure) is supposed to recycle potassium at the basolateral face of the cell allowing fueling of the sodium pump in extracellular potassium. At the apical face, the sodium is cotransported with 1 potassium and 2 chlorides by the NKCC2 cotransporter. The four sites of the NKCC2 have to be occupied to generate an electroneutral transport. As the urinary fluid is 30-fold more concentrated in sodium than in potassium, potassium ions are recycled in the urine fluid at the luminal face of the cell through the ROMK (rectified outer medullary potassium) channel, in order to provide enough potassium for a continuous activity of the NKCC2. The 2 chlorides are reabsorbed at the basolateral face of the cell by the chloride channels CLCKA and B. Those channels need to be addressed and clustered at the basolateral membrane by the barttin. The main regulator of sodium reabsorption in the Henle loop is the vasopressin (also referred as antidiuretic hormone). The asymmetry of charges in the lumen (excess of potassium) and in the interstitium (excess of chloride) generates a potential according to the 3^rd^ principle of thermodynamics (Nernst formula). The paracellular transport of cations (calcium and magnesium as well as sodium and potassium in some extent) through the intercellular space is allowed by special claudins (claudines16 and 19, also referred as paracellins) and dissipates the transepithelial potential of the Henle loop [2, 3].

**Figure 3:**
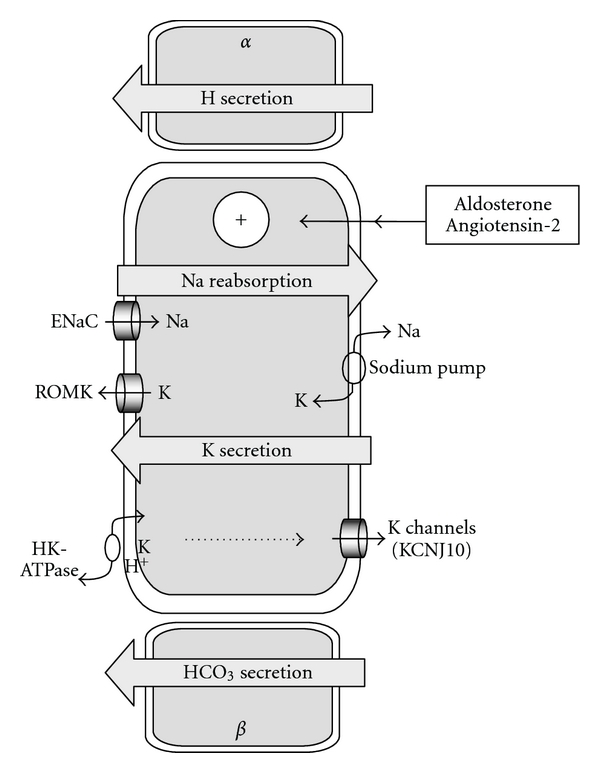
*Activation of the principal cell of the collecting duct by aldosterone and angiotensin-2*. The collecting duct is a composite structure made of 3 types of cells: the principal cell that assumes sodium and water reabsorption and potassium secretion, the *α*-intercalated cell dedicated to hydrogen secretion, and the *β*-intercalated cell dedicated to bicarbonate secretion. In the principal cell, the reabsorption of sodium is vectorized through the epithelial sodium channel at the apical side of the principal cell and the sodium pump at the basolateral side. Potassium ions are cross-transported against sodium ions by the sodium pump and secreted in the lumen by the ROMK channel that is the same ROMK channel expressed in the Henle loop. Chronic sodium depletion and the subsequent extracellular volume contraction lead to an activation of the renin-angiotensin2-aldosterone axis that leads to the enhancement of sodium reabsorption and an exacerbated cross-secretion of potassium by the principal cell. The subsequent potassium depletion leads to hypokalemia and likely induces the expression of the gastric proton-potassium pump (inhibited by omeprazole) at the apical face of the principal cell leading to an excessive secretion of hydrogen and a subsequent alkalosis [18].

Bartter syndrome is a hereditary disorder that has been characterized by the association of hypokalemia, alkalosis and the hypertrophy of the juxtaglomerular complex with secondary hyperaldosteronism and normal blood pressure [1]. By contrast, the genetic causes of Bartter syndrome primarily affect molecular structures directly involved in the sodium reabsorption at the level of the Henle loop (see the functional segmentation of sodium reabsorption in Figure 1 and [2, 3]. The ensuing urinary sodium wasting and chronic sodium depletion are responsible for the contraction of the extracellular volume, the activation of the renin-aldosterone axis, the secretion of prostaglandins, and the biological adaptations of downstream tubular segments, meaning the distal convoluted tubule and the collecting duct. These secondary biological adaptations lead to hypokalemia and alkalosis, illustrating a close integration of the solutes regulation in the tubular structures.

## 2. Primary Molecular Defects and Direct Consequences

Genome alterations leading to the Bartter syndrome affect the genes encoding different molecules involved in the sodium reabsorption at the level of the large ascending limb of the Henle loop (Figure 2). Sodium-Potassium-Chloride Cotransporter (NKCC2, gene *SLC12A1*) is responsible for Bartter type I, the inwardly rectified potassium channel (ROMK or rectifying outer medullary potassium channel, gene *KCNJ1*), for Bartter type II, the chloride channel Kb (ClCKb, gene* CLCNKB;*) for Bartter type III, the barttin (gene *BSND) *for Bartter type IV [3]. A complete deletion of *CLCNKB *gene with additional alterations of the *ClCK-A* gene leads to a severe form of Bartter syndrome similar to Bartter syndrome type IV [4, 5]. A biological phenotype of Bartter syndrome has also been reported in different damages of molecular structures that directly or indirectly affect sodium reabsorption in the Henle loop: the calcium-sensing receptor (*CaSR*, activating mutation L125P) that mainly leads to hypocalcemia and hypercalciuria, sometimes referred as type V Bartter syndrome [6], the Chloride channel-5 (ClC5) [7], and the cystinosin [8]. Gitelman syndrome, due to the alteration of the *SLC12A3* gene that encodes the Sodium chloride cotransporter in the distal convolution, is also marked by sodium wasting, hyperreninism, hyperaldosteronisme,and hypochloremic alkalosis but differs from Bartter syndrome by hypomagnesemia and hypocalciuria [3]. The basolateral potassium channel (*KCNJ10)* is the cause of the EAST syndrome (Epilepsy, Ataxia, Sensorineural deafness, and Tubulopathy) that also displays a phenotype closer from Gitelman syndrome than from Bartter syndrome [9]. The chronic use of furosemide that blocks the NKCC2 leads to an acquired biological phenotype of Bartter syndrome [10]. Nephrotoxic agents (aminoglycosides, amphotericin B, and heavy metal intoxication) have also been reported to be sometime associated with a phenotype of Bartter syndrome [11–13]. Extrarenal loss of sodium chloride by the gastrointestinal tract (congenital chloride diarrhea) or by the skin (cystic fibrosis) may also display a biological phenotype of Bartter syndrome but these patients that have a normal renal tubular function differed from true Bartter syndrome by a very low excretion fraction of sodium [14, 15]. 

### 2.1. Urinary Sodium Wasting

Assuming a glomerular filtration rate of 100 mL/min and a serum sodium concentration of 140 mmol/L, about 20 mol/day of sodium are filtered everyday, equivalent to the amount of 1.2 kg of salt. Under physiological conditions, renal tubules are capable of reabsorbing 99 to 100% of filtered sodium and water. The primary consequence of a defect in the Henle loop is a failure to reabsorb 30% of the filtered sodium. The massive amount of sodium that is delivered downstream to the end of the Henle loop exceeds the possibility of compensation by the distal convoluted tubule and the collecting duct and leads to urinary sodium wasting [3].

### 2.2. Urinary Concentration and Dilution Defect

A second consequence of the defect of Henle loop is the abolition of the corticopapillary osmolar gradient that prevents the ability to concentrate the final urine over the plasma osmolality. In addition, patients display a partial impairment in diluting urine far below the plasma osmolality while half of the capacity of diluting urine is due to the anhydrous reabsorption of sodium chloride in the Henle loop, and the second half is due to the anhydrous reabsorption of sodium chloride in the distal convoluted tubule [16]. 

### 2.3. Hypercalciuria

Urinary calcium wasting is due to the abolition of the transtubular potential, the subsequent inability to passively drive divalent cations through the intercellular space in the Henle loop (see Figure 2) and the patent failure of downstream adaptation. The chronic loss of calcium is frequently responsible for an increased bone resorption and a decrease of bone mineralization that is proportional to the level of urine calcium [17]. Calcium supplementation may prevent these bone alterations.

## 3. Secondary Biological Adaptations

### 3.1. Hyperreninism

The extracellular volume is a sodium salt solution that is closely maintained to a steady-state osmolality of 300 mosmol/Kg. Chronic sodium depletion subsequently leads to a contraction of the extracellular volume. Hypotension, failure to thrive, and at the biological level, either an increased concentration of the total plasma protides or a high hematocrit are the common signs of extracellular volume contraction [19, 20]. Chronic hypovolemia results in a stimulation of the renin axis and enhances the production of angiotensin-2 that stimulates the sodium reabsorption in the principal cell of the collecting duct (Figure 3) [21].

### 3.2. Hyperaldosteronism

Angiotensin-2 directly increases the secretion of aldosterone by the *granulosa* of the adrenal glands [22]. Aldosterone adds to the stimulation of the sodium reabsorption in the collecting duct and also likely stimulates those in the distal convoluted tubule [23]. Hyperaldosteronism may be dampened, relatively to a high renin stimulation, by hypokalemia and potassium depletion that directly interfere with aldosterone secretion in the *granulosa* [22].

### 3.3. Hypokalemia

As in any case of hyperaldosteronism, the exacerbation of the sodium reabsorption is closely associated to an exacerbation of the potassium secretion due to the stimulation of the crossed transport activity of sodium and potassium by the sodium pump. Consistently, hyperaldosteronism and urinary potassium wasting improve when sodium supplementation is increased [24]. Conversely, hypokalaemia and potassium depletion may improve with angioconvertase inhibitors, likely through a decrease of production of angiotensin-2 and the deactivation of the collecting duct [25]. Accordingly, patients treated with enalapril had a fall in blood pressure and a decrease in glomerular filtration rate [25].

### 3.4. Alkalosis

The mechanism of alkalosis might be explained by an effect of aldosterone on the expression of the proton ATPase located in the *α*-intercalated cell but, *α*-intercalated cell does not express the mineralocorticoid receptor [26, 27]. A second possibility is that the potassium depletion might be responsible for a *de novo* expression of the gastric proton-potassium ATPase (sensitive to omeprazole) at the luminal face of the principal cell in order to reabsorb a part of the secreted potassium. For each potassium ion that is internalized, 1 hydrogen is extruded in the urine by the proton potassium ATPase, generating alkalosis [18, 28]. This adaptation that has been fully demonstrated in rat models of potassium depletion might also apply to humans.

### 3.5. Complications of Potassium Depletion

Hypokalemic periodic paralysis and rhabdomyolysis has been reported for hypokalaemia below 2 mmol/L and fully resolved following the reload of the system with potassium chloride [29, 30]. Prolonged QT interval has been evidenced with hypokalemia below 2 mmol/L, and subsequent aborted sudden cardiac death may occur [31, 32]. Potassium depletion also induces coronary microvascular and myocardial defects during exercise in patients with Bartter syndrome [33].

### 3.6. Polyuria

The mechanism of polyuria in Bartter syndrome has to be considered differently in the antenatal period and after birth. In the antenatal period, the final urine delivered by a normal fetus is hypoosmolar suggesting that the water reabsorption in the collecting duct is not yet matured before birth while the anhydrous reabsorption of sodium chloride is fully functional in the Henle loop [34, 35]. In the amniotic fluid, the fetal urine is balanced with the fetal plasma that circulates in the fetal membranes (amnion and chorion) [34]. In physiological conditions, the difference of the osmotic pressure between the amniotic fluid and the fetal plasma strongly favors a water outflow from the amniotic fluid into the capillaries of the fetal membranes [36]. Indeed, 5 types of aquaporins (AQP-1, -3, -4, -8, -9) are strongly expressed in the amnion and the chorion [37]. In fetus with Bartter syndrome, the amniotic fluid is fed with the fetal urine that is released at the end of the proximal convoluted tubule and not modified in the Henle loop. In these conditions, the fetal urinary osmolality is equal to those of the fetal plasma, likely preventing any water transfer from the amniotic fluid into the fetal plasma and leading to a polyhydramnios. The concentration of solutes remains balanced in the amniotic fluid while the total protein concentration (the solid phase) is diluted [38]. After birth, the polyuria is supposed to be generated by the addition of the inability of the kidneys to concentrate the final urine over the plasma osmolality and the increase of excreted osmoles due to the urinary sodium and potassium wasting.

### 3.7. Hyperprostaglandinuria

Hyperprostaglandinuria is not specific of Bartter syndrome and has been either reported in sodium depletion without Bartter syndrome [39, 40] or in water deprivation [41–43]. Tissular secretion of PGE_2_ is stimulated by the vasopressin [44] either secondary to hypovolemia due to a sodium depletion or hypernatremia. In the kidney, cyclooxygenase-1 (COX1) is highly expressed in the collecting duct, and cyclooxygenase-2 (COX2) is expressed predominantly in the renal medullary interstitial cells, in the cortical thick ascending limb and in the cells associated with the macula densa. In addition, prostaglandin E synthase (PGES1) is highly expressed in the collecting duct and also detected in the thick ascending limb and the macula densa [45].

## 4. Specificities according to the Molecular Defect

### 4.1. SLC12A1 (NKCC2)

Mutations of *SLC12A1* gene have been associated with the most typical form of antenatal Bartter syndrome featuring early onset of polyhydramnios, neonatal polyuria, and dehydration requiring massive amount of water and sodium supplementation in the first days of life, detectable nephrocalcinosis on renal ultrasound within the first month, and high plasma level of renin and secondary hypokalemic alkalosis following the first days of life [46, 47]. A delayed onset in life has also been exceptionally reported in cases with a residual function of the NKCC2 cotransporter [48].

### 4.2. ROMK (KCNJ1)

Mutations in the *KCNJ1* gene display the same clinical phenotype as described in the NKCC2 section, but transient hyperkalemia and acidosis are observed during the first month of life [46, 47, 49]. Kalemia secondarily decreases along the course of the disease, but hypokalemia frequently failed to develop in these patients [47, 49]. A delayed onset in life has also been exceptionally reported in cases with a residual function of the ROMK channel [50].

### 4.3. ClCK-b (CLCNKB)

Mutations in the *CLCNKB* gene give the most variable clinical phenotypes from early polyhydramnios and severe neonatal polyuria [46, 47] to classic Bartter syndrome with a delayed diagnosis in childhood and failure to thrive [51] and to the phenotype of Gitelman syndrome without polyuria [52]. Hypercalciuria may lack in some patients with *CLCNKB* mutations [46].

### 4.4. Barttin (BSND)

Barttin mutations are marked by deafness [53]. Most of the patients have a severe tubular disease, but a few adults have been recognized on deafness with a mild renal phenotype [54]. Hypercalciuria may also lack in a set of patients with *BNSD* mutations [46].

## 5. Treatment

Undelayed management of neonates with fully symptomatic antenatal Bartter syndrome is a necessity. Prenatal diagnosis relies on the occurrence of polyhydramnios without any classical cause of polyhydramnios (mainly maternal diabetes and fetal gastrointestinal tract malformation). The analysis of sodium, potassium, and chloride concentration relatively to those of total protein and alphafetoprotein in the amniotic fluid (the Bartter Index) is a reliable tool to confirm the diagnosis when familial history is lacking [38]. 

### 5.1. Sodium Chloride and Water

According to the physiopathology of the disease, the primary treatment of Bartter syndrome, at least in the neonatal period and in childhood, is the supplementation in sodium chloride. Data are scarce but some reports show that the amounts of sodium chloride needed to balance the extracellular volume, and prevent dehydration and hypovolemia to limit the stimulation of the renin axis, may reach 50 mmol/Kg/day in neonates [46, 55, 56]. Polyuria may occur immediately after birth prior to any sodium supplementation and requires water inputs that have sometimes to be rapidly increased up to 500 mL/Kg/day within the first days of life [55, 56]. Secondarily, continuous supplementation with water and sodium chloride either through a nasogastric tube or a gastrostomy may be necessary to improve growth in weight and length during the first months or years of life [57, 58]. A free access to sodium chloride and water when children gain food autonomy is as much as important that a free access to water for the children that are affected with a nephrogenic diabetes insipidus. Intravenous infusions of sodium salt solutions are necessary to prevent life-threatening dehydration in case of vomiting or diarrhea.

### 5.2. Potassium Chloride

Sodium chloride supplementation should be the best way to preserve children from hypokalemia and potassium depletion through the control of the extracellular volume and the renin-aldosterone axis. [59] Nevertheless, in some patients, the amounts of sodium chloride that would balance the extracellular volume and the renin system are so elevated that they are not acceptable by the patient. A potassium-rich diet and potassium chloride supplementation are subsequently needed.

### 5.3. Indomethacin

Indometacin has been used either prior to birth to prevent the recurrence of a polyhydramnios or after birth to limit the polyuria [60, 61]. In the fetus with a Bartter syndrome, the amount of urine that is delivered at the end of the proximal convoluted tubule is directly proportional to the glomerular filtration rate. Therefore, the effect of indomethacin in preventing the recurrence of polyhydramnios following a drain is likely due to its action on glomerular vasculature, the reduction of the fetal glomerular filtration rate, and the subsequent reduction of fetal urine output [62]. After birth, prostaglandins are paradoxically potent inhibitors of the sodium reabsorption in the collecting duct through their specific receptor EP_1_ and EP_2_ [45, 63]. In addition, inhibition of prostaglandin synthesis during fasting in healthy man increases renal sodium absorption through a possible direct regulation of the epithelial sodium channels [64]. In the same time, prostaglandins are likely to be responsible for a decrease of the expression of aquaporin-2 and the *V*_2_ receptor resulting in a decrease of water permeability in the collecting duct [65] that is supposed to amplify the polyuria. Therefore, the beneficial effect of indomethacin on urinary sodium wasting and polyuria might be better figured out by the inhibition of the prostaglandins pathway on tubular structures than by a mild or a moderate decrease of the glomerular filtration rate [66, 67]. However, prescribers must be aware that necrotizing enterocolitis and death have been reported following the use of indomethacin in the neonatal period [55, 58, 68–70].

### 5.4. Potassium-Sparing Drugs

Many drugs are susceptible to block the secretion of potassium in the collecting duct and to prevent potassium depletion. Those that have been used in Bartter syndrome are modamide, the antagonist of the Epithelial Sodium Channel, spironolactone, the antagonist of mineralocorticoid receptor, and angiotensin-converting enzyme inhibitors. Unfortunately, all these drugs impair the stimulation of the sodium reabsorption in the collecting duct and are susceptible to worsen the urinary sodium wasting and the chronic contraction of the extracellular volume [25, 71, 72].

## 6. Unresolved and New Questions

### 6.1. Cysts

The development of renal cysts have been reported in patients with Bartter syndrome, of whom one had a mutation in the *CLCNKB* gene [61, 73]. The mechanism remains unveiled.

### 6.2. Chronic Renal Failure

Renal fibrosis has been reported in a series of 12 patients, of whom four had a decreased glomerular filtration rate. No statistical correlations could be established between the indomethacin dose and the percentage of altered interstitial surface area [74]. By contrast, significant relationships have been evidenced between the severity of urinary sodium wasting and the level of the glomerular filtration rate suggesting that the extent of renal fibrosis might be related to the chronic contraction of the extracellular volume and hypovolemia. Consistently, the development of chronic renal failure is a frequent feature in patients with a barttin mutation in parallel to severe chronic extracellular dehydration [53, 75]. In addition, chronic renal failure has also reported in patients who developed proteinuria and lesions of focal segmental glomerulosclerosis superimposed to a Bartter syndrome [76, 77]. Finally, end stage renal failure is rare but has been yet reported [78].

### 6.3. Secondary Diabetes Insipidus

Hypernatremia with spontaneous diluted urine below 100 mosmol/Kg and failure to concentrate urine over 200 mosmol/Kg has been reported without any mutation in the genes of the *V*_2_ receptor and aquaporine-2 [57]. Diluted final urine may be paradoxical in Bartter syndrome while the Henle loop is a major site to dilute urine. One should recall that the first half of the distal convolution is also water impermeable while aquaporine-2 is never coexpressed along with the sodium-chloride transporter [79] and that the distal convolution may consequently account for by a nonnegligible part of the diluting urine capacity [80]. Nevertheless, the failure of the principal cell of the collecting duct to appropriately transport water when strongly and continuously activated by angiotensin-2 and aldosterone remains to be understood.

### 6.4. Cholelithiasis

A case of cholelithiasis has been reported recently and attributed to alkalosis and dehydration that favour precipitation of calcium carbonate in biliary ducts [81].

## 7. Conclusion

In conclusion, renal physiology allows a rational approach to understand the biological features of Bartter syndrome and supports sodium chloride supplementation as the most appropriate treatment to control both the extracellular volume and the potassium depletion. On a basic point of view, it is noteworthy that the preservation of the sodium pool (and the balance of the extracellular volume) is a higher priority of the system than the protection against the potassium depletion, itself a priority upon the hydrogen balance.
